# A Randomized Controlled Clinical Trial of Lifestyle Intervention and Pioglitazone for Normalization of Glucose Status in Chinese with Prediabetes

**DOI:** 10.1155/2022/2971382

**Published:** 2022-01-06

**Authors:** Yingying Luo, Hongyuan Wang, Xianghai Zhou, Cuiqing Chang, Wei Chen, Xiaohui Guo, Jinkui Yang, Linong Ji, Sanjoy K. Paul

**Affiliations:** ^1^Department of Endocrinology and Metabolism, Peking University People's Hospital, Beijing, China; ^2^Department of Epidemiology and Biostatistics, School of Public Health, Peking University Health Science Center, Beijing, China; ^3^Institute of Sports Medicine, Peking University Third Hospital, Beijing, China; ^4^Department of Parenteral and Enteral Nutrition, Peking Union Medical College Hospital, Beijing, China; ^5^Department of Endocrinology and Metabolism, Peking University First Hospital, Beijing, China; ^6^Department of Endocrinology and Metabolism, Beijing Tongren Hospital, Capital Medical University, Beijing, China; ^7^Melbourne EpiCentre, University of Melbourne and Melbourne Health, Melbourne, Australia

## Abstract

**Aims:**

Prediabetes has been proved as an important risk factor of both diabetes and cardiovascular disease (CVD). Previous studies have shown that both lifestyle intervention and pioglitazone may delay the development of diabetes in patients with prediabetes. However, no study has ever explored whether these interventions could revert prediabetes to normal glycemic status as the primary outcome. Interventions that may revert prediabetes back to normal glucose status would be of great clinical importance.

**Materials and Methods:**

We conducted a randomized, multicenter, 2 × 2 factorial designed study to examine whether intensive lifestyle intervention and/or pioglitazone could revert prediabetes to normal glucose tolerance. The participants were followed up for three years unless they reverted to normal glucose state or developed diabetes at the annual oral glucose tolerance test (OGTT). Reversion to normal glucose tolerance was confirmed on the basis of the results of OGTT.

**Results:**

In our study, 1945 eligible patients were ultimately randomized into four groups. In this three-year follow-up study, overall, 60.0%, 50.3%, 56.6% and 65.1% reverted back to normoglycemic state over 3 years of follow-up in the conventional lifestyle intervention plus placebo, intensive lifestyle intervention plus placebo, conventional lifestyle intervention plus pioglitazone, and intensive lifestyle intervention plus pioglitazone groups, respectively. Compared to the conventional lifestyle intervention plus placebo group, all the other three groups did not show any significant benefit in terms of reverting back to normoglycemic state.

**Conclusion:**

In our study, for patients with prediabetes, neither intensive lifestyle intervention nor pioglitazone had led to a higher reversion rate to normal glucose state. *Trail registration.*http://www.chictr.org.cn: ChiCTR-PRC-06000005.

## 1. Introduction

Diabetes is one of the major components of the burden of disease in China, while a significant proportion of the population has prediabetes. The most recent data showed that among adults in China, the estimated overall prevalence of diabetes was 10.9% and that for prediabetes was 35.7% [1]. Patients with prediabetes are not only at high risk to develop diabetes but also the high risk population to develop cardiovascular disease (CVD) [2, 3].

Da Qing study was the first intervention study for prediabetes all over the world, which was also a prevention study in Chinese population [4]. After a 23-year follow-up, it showed that the majority of deaths (74.7%; 130 of 174) occurred in those who developed diabetes. Progression to type 2 diabetes was associated with a 73% higher risk of death in this study. In China, the risk of mortality is increased in people with prediabetes; this excess risk might be explained by the development of type 2 diabetes [5]. Numerous clinical trials had showed that early intervention based on individualized prevention model may be beneficial in delaying the progression to type 2 diabetes in high-risk populations [6].

It is possible that interventions which could revert prediabetes to normal or could delay the development of diabetes may also be effective to decrease the risk of developing long-term complications of hyperglycemia or death. During the last 30 years, many studies have shown that lifestyle modification [4, 7–13]; the use of metformin [14], acarbose [10, 15], and thiazolidinediones [11, 12, 16]; and bariatric surgery may delay the progression of type 2 diabetes in patients with impaired glucose tolerance [17].

However, no study has explored whether intervention could revert prediabetes to normal glycemic status as the primary outcome. The previous studies were all aimed at evaluating the efficacy of intervention on preventing diabetes in patients with impaired glucose tolerance (IGT), rather than prediabetes. We undertook the present study to evaluate whether lifestyle intervention with or without pioglitazone could revert prediabetic state back to normal glycemia over a 3-year period in patients with prediabetes.

## 2. Materials and Methods

### 2.1. Study Design

As described in our previous publication [18], Beijing Prediabetes Reversion Program (BPRP) is a prospective, multicenter, randomized, double-blinded, and placebo-controlled clinical trial, based on a 2 × 2 factorial design. Patients with prediabetes were randomized into four groups: conventional lifestyle intervention plus placebo, conventional lifestyle intervention plus pioglitazone hydrochloride 30 mg daily, intensive lifestyle intervention plus placebo, and intensive lifestyle intervention plus pioglitazone hydrochloride 30 mg daily.

### 2.2. Participants

As published elsewhere [18], individuals who were diagnosed as prediabetes based on previous oral glucose tolerance test (OGTT) were eligible for screening. We recruited male and female patients who were between 25 and 70 years old and had prediabetes (defined as fasting plasma glucose ≥ 6.1 mmol/L (110 mg/dL) and <7.0 mmol/L (126 mg/dL), meanwhile 2hPG < 7.8 mmol/L (140 mg/dL) or FPG < 7.0 mmol/L (126 mg/dL), meanwhile, 2hPG ≥ 7.8 mmol/L (140 mg/dL) and <11.1 mmol/L (200 mg/dL) during a single oral glucose tolerance) and a body mass index (BMI) between 22 and 35 kg/m^2^. Informed consent forms were obtained before the individuals could participate in any screening procedures. Eligible participants were then randomized into one of the four arms of the study. The detailed inclusion and exclusion criteria have been published previously [18].

The first participant was screened in March 2007. The enrollment of 1945 participants was completed in March 2011. Participants were followed until they reverted to normal glucose level or developed diabetes, withdrew from the study, were lost to follow-up, or completed the end of the study. There were 4397 individuals who met the screening criteria and were screened. Among these individuals, 1388 participants were identified normal glucose tolerance and 975 participants had diabetes. Finally, 2034 (46.3%) were confirmed to have prediabetes. Among 4397 screened population, the mean ± SD age and BMI were 52 ± 15 years and 26.1 ± 3.3 kg/m^2^, respectively. Following the inclusion and exclusion criteria, 1954 eligible patients were randomized into four groups with equal proportion. The mean age of the participants was 53 ± 10 years old, and the median (Q1, Q3) of BMI and HbA1c was 26.0 (23.9, 28.2) kg/m^2^ and 5.8 (5.6, 6.1)%, respectively. 85% of the participants were IGT, and 15% were IFG.

### 2.3. Interventions

In our previous publication, we have described the intervention in detail [18]. In brief, all the participants were recruited and followed up in the outpatient clinic. The follow-up of the study was three years. There were 19 scheduled visits during the three-year follow-up. To avoid the bias, in all four groups, study visits were scheduled for every 2-3 weeks during the first 13 weeks and every 13 weeks thereafter.

In the intensive lifestyle intervention group, after randomization, educational courses were provided to the participants at each visit and the investigators would prescribe an individualized lifestyle prescription for them at each visit according to their body weight and lifestyle diary with decision support software we made particularly for this study.

The software was built based on the dietary and exercise recommendations from dietary guidelines for Chinese residents and guideline for prevention and treatment of type 2 diabetes. In the intensive lifestyle intervention group, participants were suggested to have at least 150 minutes of moderate intensity (3-6MET) of aerobic exercise every week and were encouraged to have 180-300 minutes per week. Besides the aerobic exercise, participants were also encouraged to have some resistant exercise every week.

In the dietary instruction, the aim was to achieve negative balance of energy intake. The Harris-Benedict formula was used to calculate the total energy intake [19]. A 300 kcal deficit was provided for overweight population, and a 500 kcal deficit was provided for obese population.

The weight loss goal was 5-10% reduction (or 2–4 kg per week) in body weight from baseline for those who were obese or overweight. At each study visit, the dietary intake component from the lifestyle diary and the body weight was used to generate the automated lifestyle prescription from the software, with the option for manual changes in the prescription if deemed necessary.

In the conventional lifestyle intervention group, participants were provided usual lifestyle education at both baseline and annual visits; they would not receive any individualized consultation. They would only receive general information on healthy lifestyle and would not receive lifestyle evaluation or lifestyle-related prescription. To avoid the contamination between groups, all four groups were designed with similar visit schedule.

Among participants who received pioglitazone, the dose of the medication (30 mg/day) would not change throughout the follow-up period. The active drug and matched placebo were manufactured by Beijing Taiyang Pharmaceutical Company.

At annual visit, participants who were identified to have reverted to normal glucose state were requested to stop the medication and were invited for an OGTT two weeks after the last visit. This procedure was followed for those participants who remained prediabetic during the course of 3 years of follow-up. Those who were identified to have developed diabetes after 2 weeks of washout period were advised to seek standard care for diabetes. At the end of follow-up, all participants were advised to follow standard lifestyle modification and those who still had elevated glucose level were transferred to outpatient clinic.

### 2.4. Objectives

This study was used to examine whether intensive lifestyle intervention and/or pioglitazone 30 mg once daily would increase the reversion rate of patients with prediabetes to normal glycemia, compared to conventional lifestyle intervention only.

### 2.5. Primary and Secondary Outcomes

As mentioned in our previous publication [18], the primary aim of the study was to evaluate the proportions of participants who reverted to normal glucose state during follow-up. The normal glucose level were was defined by OGTT glucose level with fasting plasma glucose less than 6.1 mmol/L and 2-hour postchallenge glucose less than 7.8 mmol/L. The secondary outcomes of the study included the following: (1) incidence of type 2 diabetes; (2) time to achieving normal glucose level; (3) change in HbA1c; (4) change in body weight and waist circumference; (5) changes in blood pressure, LDL-cholesterol, and HDL-cholesterol and triglyceride; (6) changes in adiponectin, hsCRP, and insulin and C-peptide at fasting and postchallenge; (7) change in urine albumin-creatinine ratio and serum creatinine; (8) composite of the incidence of at least one of the events, heart failure, nonfatal myocardial infarction, nonfatal stroke, or all-cause mortality; and (9) quality of life.

### 2.6. Sample Size

As described in our previous publication [18], we calculated that the sample size of this study would be 2000 participants (500 in each group). With this sample size, it could provide a 90% power with 5% type 1 error to detect a 10% relative increase in the rate of primary outcomes among participants assigned to intensive lifestyle intervention compared with conventional lifestyle intervention group under the following assumptions:

1. 35.3% for the conventional lifestyle plus placebo, 44.3% for the conventional lifestyle plus pioglitazone, 45.3% for the intensive lifestyle plus placebo, 54.3% for the intensive lifestyle plus pioglitazone would be reverted to normal glucose
2. Participants would be recruited in half a year
3. Up to 30% of the participants might be lost of follow up during the study period

### 2.7. Randomization

We have mentioned in our previous publication [18] that randomization was undertaken by an independent statistician using a computer-generated random sequence and was performed as block randomization. The allocation ratio in four groups was 1 : 1 : 1 : 1. Each study site would receive sealed envelopes for randomization of the participants. Both the participants and healthcare providers were blinded by the medication, while they were open to the lifestyle intervention.

### 2.8. Statistical Methods

Basic statistics are presented by number (%), mean (SD), or median (Q1, Q3). The primary and secondary outcomes of the study were evaluated following the intention to treat approach, with additional supporting analyses based on the per protocol population.

To evaluate the proportion of people reverting to normoglycemic state and the proportion of people developing T2DM during the three years of follow-up in the intensive lifestyle intervention plus placebo, conventional lifestyle intervention plus pioglitazone, and intensive lifestyle intervention plus pioglitazone groups, compared to conventional lifestyle intervention plus placebo group, the discrete-time survival regression model with development of diabetes being the competing risk while evaluating the risk of reverting to normoglycemia. The hazard ratios and 95% CI were estimated. As an additional analysis, the logistic regression was also used, and the odds ratio (95% CI) was estimated with the significance level based on Bonferroni corrections. Changes in anthropometric, clinical and laboratory based secondary outcome measures at 1, 2, and 3 years of the study were estimates and presented as mean change (95% CI).

## 3. Results

### 3.1. Participants' Flow

**Figure 1:**
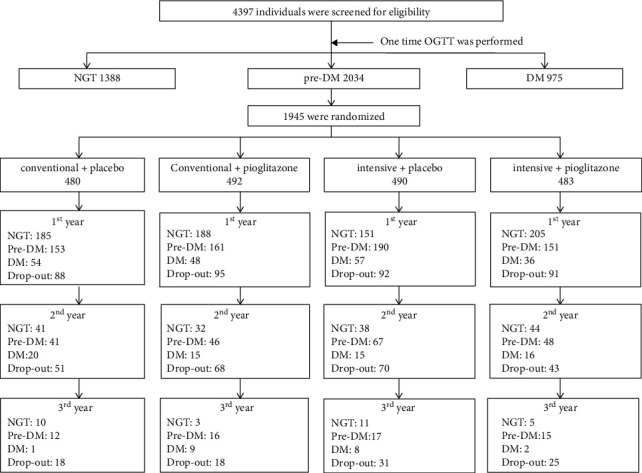
Participant flow.

In our study, 4397 individuals who met the screening criteria were screened. Among these individuals, 2034 (46.3%) were finally identified to have prediabetes. Following the inclusion and exclusion criteria, 1945 eligible patients were ultimately randomized into four groups (Figure 1). The baseline characteristics of the participants are described in Table 1. At the end of the first year, 366 participants were lost in follow-up. At the end of the second and third years, 232 and 92 participants were lost in follow-up, respectively. The overall dropout rate was 35% during three years of follow-up. With the software built for the lifestyle intervention prescription, we also evaluated the compliance of the lifestyle intervention in two intensive arms. The percentage of good compliance with diet was 18%, 20%, and 19% for the first, second, and third years, while the percentage of good compliance with exercise was 65%, 67%, and 66% for the first, second, and third years (supplement Figure 1).

### 3.2. Primary Outcome

**Figure 2:**
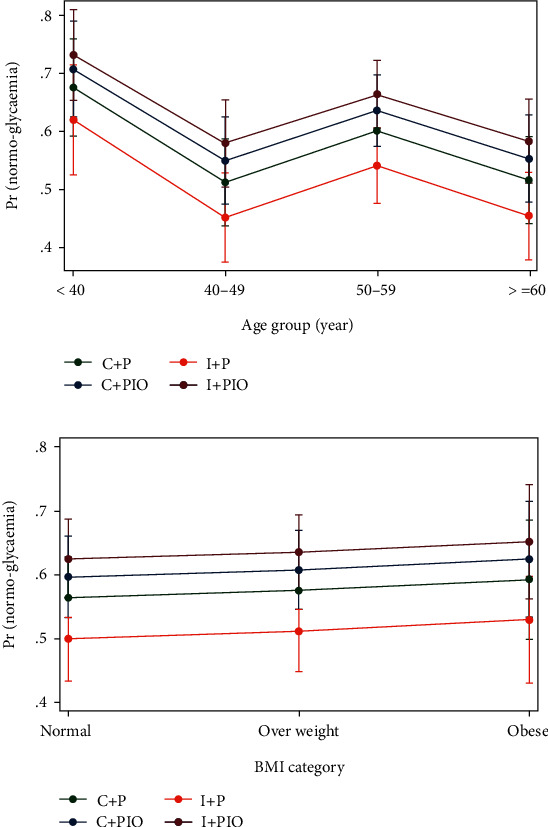
Estimated probability of reaching normoglycemia during three years of follow-up by the treatment group: (a) separately for different age groups at baseline and (b) separately for different BMI categories at baseline.

The proportions of individuals reverting to normoglycemia and diabetes at 1, 2, and 3 years of follow-up and overall during 3 years of follow-up are presented in Table 2. Within 1 year of follow-up, 38.5%, 30.8%, 38.2%, and 42.4% reverted to the normoglycemic state in the conventional lifestyle intervention plus placebo, intensive lifestyle intervention plus placebo, conventional lifestyle intervention plus pioglitazone, and intensive lifestyle intervention plus pioglitazone groups, respectively. Overall, 60.0%, 50.3%, 56.6%, and 65.1% reverted back to normoglycemic state over 3 years of follow-up in these treatment groups, respectively. Compared to the conventional lifestyle intervention plus placebo group, all the other three groups did not show any significant benefit in terms of reverting back to normoglycemic state. We further divided the participants into different age groups and BMI groups. We see that the proportion of participants who reverted to normal glycemia state was highest in the youngest group (age < 40 years) and obese group (BMI ≥ 30 kg/m^2^) (Figure 2).

### 3.3. Secondary Outcomes

Compared to the conventional lifestyle intervention plus placebo group, individuals in the intensive lifestyle intervention plus pioglitazone group had 45% (95% CI of HR: 0.32, 0.90, *p* = 0.024) reduced risk of developing diabetes, while the likelihood of developing diabetes was similar in the other two study groups. The changes in the secondary outcome measures during 3 years of follow-up from baseline are presented in Table 3. The changes in the fasting and postprandial glucose levels were not different between the treatment groups (*p* > 0.05), while we observed statistically significant reduction in postprandial glucose levels in the conventional lifestyle intervention plus placebo group (95% CI -2.0, -0.12 mmol/L) and intensive lifestyle intervention plus pioglitazone group (95% CI: -0.81, -0.62 mmol/L) (*p* < 0.01 in both groups). A marginal increase in the HbA1c level was observed in all groups (range of 95% CI: 0.05–0.46%), while there was no difference between the groups.

The observed differences in the changes in body weight and waist circumference were not different between the groups. The changes in blood pressure, lipids, and other cardiovascular and renal risk factors were also not different between the treatment groups. A statistically significant reduction in the levels of C-peptide was observed in all groups (range of 95% CI: -0.25, -0.72), while such reduction was not different between the groups.

### 3.4. Safety Analysis

The adverse events, considered relevant by the investigators, are listed in Supplement Table 1. The most common adverse event was edema without any difference among four groups.

## 4. Discussion

BPRP is the first study to evaluate whether lifestyle intervention and/or pioglitazone could revert prediabetic state back to normoglycemia in Chinese population.

In our study, the reversion rates to normal glucose state were similar among all four groups. This is inconsistent with most of the previous publications. In the most previous prevention study, there always had a benefit from either intensive lifestyle intervention or a certain kind of antidiabetic drugs. Since the primary endpoint was the prevention of diabetes in almost all the studies, there were only a few studies that had reported the reversion rate of normal glucose state. DPP study was one of the most famous diabetes prevention study with lifestyle intervention. After the 3 years' follow-up, the reversion rate in the control group and lifestyle intervention group were around 25 percent and 35 percent, respectively [14], while the overall reversion rate in our study ranged from 50 percent to 65 percent, which was even much higher than that in the intervention group in DPP study. If we take a look at the data in the ACT NOW study, which was also a diabetes prevention study with pioglitazone [12], the conversion to normal glucose tolerance occurred in 48% of the patients in the pioglitazone group and 28% of those in the placebo group. The overall conversion rate in any one of the groups in our study was higher than that reported in the ACT NOW study.

When comparing the incidence of diabetes from our study with other studies, we can see that the incidence of diabetes in the control group in our study is much lower than that shown in the control group in the previous study. The incidence of diabetes in our study ranged from 11.18 percent to 16.33 percent among all four groups. DPP study was one of the most famous diabetes prevention study. In that study, there were 28.9% in the placebo group and 14.4% in the lifestyle intervention group developed diabetes at three years. If we look at the DPS study, which had a similar follow-up duration with our study, we also found that after the mean 3.2 years follow-up, the cumulative incidence of diabetes was 11 percent in the intervention group, which was similar with our study and 23 percent in the control group, which was much higher than our study [20].

Therefore, we believe that the most possible reason for our negative result is because the conversion rate to normal glucose state in the control group in our study is relatively high. When we designed this study, we designed an exact same follow-up plan for both the conventional lifestyle intervention group and the intensive lifestyle intervention group. This may lead to a minimized difference between the effects on the change of the glucose level from conventional or intensive lifestyle intervention group. Besides this, nowadays, there is also more and more educational information from public media that the participants may receive. So, the participants in our study were totally different with those who were recruited in the Da Qing study twenty years ago.

From the baseline data, we can also see that the BMI of the participants in our study was far lower that shown in many diabetes prevention studies in the Western country. The mean BMI of the participants in our study was only 26 kg/m^2^, and nearly 40% of the participants had a normal BMI. Therefore, lifestyle intervention may not have as much effect as it does for those who were very obese. However, if we look at the change of the body weight at the end of the study, we can still see that there was a 2.21 kg decrease of the body weight in the conventional lifestyle intervention plus placebo group. This may be another possible explanation for the negative result of the study.

There are some limitations of our study. First of all, compliance to the diet intervention in the intensive lifestyle intervention group was relatively poor. However, we had a mix of secondary- and tertiary-level hospitals, with likely differences in the levels of engagement and experience among study nurses and clinicians. Secondly, the dropout rate was 5% higher than assumed, although still maintaining the statistical power to 85%. The intensity of the lifestyle intervention and the resource for the investigator were much less compared to the previous studies. This may lead to the higher dropout rate and nonideal compliance. However, these limitations are unlikely to adversely affect the robustness of the study results. Our study should be considered a pragmatic clinical trial, reflecting the population level scenario in terms of adherence to medication and other lifestyle interventions. Therefore, the findings of this study may provide more evidence-based information to support our daily practice. Lastly, the participants in our study were all from Beijing area and almost all of them were of Han ethnicity. Therefore, from our study, it would be difficult to evaluate how the regional habit and ethnical habit might affect the glucose outcome among patients with prediabetes.

Recently, a paper named “Dubious Diagnosis” was published in Science [21], arguing that a war on ‘prediabetes' has created millions of new patients and a tempting opportunity for pharmaceutical companies and questioned the resource allocation principle for the effective management of prediabetes. Our study answered some of important global questions in relation to management of prediabetes. Clearly, while a significant number of people revert back to normoglycemia from prediabetes with or without active intervention (s), there is also a large high-risk prediabetes population to a focus on.

## 5. Conclusion

BPRP was the first study to determine if lifestyle modification and/or pioglitazone could revert prediabetic state to normoglycemia in Chinese population. There was not any significant increase of the reversion rate from both intervention strategies. However, this makes us reconsider the prevention policy nowadays in China. We may need some more feasible method to manage prediabetes in the developing country such as China in this era of information explosion.

## Figures and Tables

**Table 1:** Baseline characteristics of the participants by study groups.

	Conventional lifestyle+placebo	Intensive lifestyle+placebo	Conventional lifestyle+pioglitazone	Intensive lifestyle+pioglitazone
*N*	480	490	492	483
Male	229 (48)	167 (34)	221 (45)	207 (43)
Age (year)	53 (45, 59)	53 (47, 59)	53 (45, 59)	53 (46, 60)
<40 years	68 (14)	53 (11)	55 (11)	61 (13)
40-49 years	116 (24)	112 (23)	124 (25)	110 (23)
50-59 years	192 (40)	213 (43)	206 (42)	191 (40)
≥60 years	104 (22)	112 (23)	107 (22)	121 (25)
Ethnicity				
Han	463 (97)	467 (96)	471 (97)	460 (96)
Other	15 (3)	21 (4)	16 (3)	20 (4)
Current or ex-smokers	112 (23)	90 (18)	122 (25)	112 (23)
BMI (kg/m^2^)	26 (24, 28)	26 (24, 28)	26 (24, 28)	26 (24, 28)
Normal: BMI < 25	192 (40)	183 (37)	193 (39)	177 (37)
Overweight: 25 ≤ BMI < 30	224 (47)	242 (49)	240 (49)	253 (52)
Obese: BMI ≥ 30	63 (13)	65 (13)	59 (12)	53 (11)
Waist-hip ratio	0.89 (0.85, 0.94)	0.89 (0.84, 0.92)	0.89 (0.85, 0.93)	0.89 (0.85, 0.93)
Systolic blood pressure (mmHg)	120 (110, 130)	120 (110, 130)	120 (113, 130)	120 (110, 130)
Diastolic blood pressure (mmHg)	79 (70, 81)	77 (70, 80)	78 (70, 82)	78 (70, 80)
Fasting plasma glucose (mmol/L)	6.0 (5.4, 6.4)	6.0 (5.5, 6.4)	6.0 (5.5, 6.4)	6.0 (5.5, 6.4)
2-hour plasma glucose (mmol/L)	8.8 (8.1, 9.9)	9.0 (8.1, 9.9)	8.9 (8.1, 9.8)	8.9 (8.0, 9.9)
HbA1c (%)	5.8 (5.6, 6.0)	5.8 (5.5, 6.1)	5.8 (5.6, 6.1)	5.8 (5.6, 6.0)
Prediabetes state				
IGT	408 (85)	424 (87)	413 (84)	409 (85)
Isolated IFG	72 (15)	66 (13)	79 (16)	74 (15)
Total cholesterol (mmol/L)	4.8 (4.2, 5.6)	4.9 (4.2, 5.4)	4.9 (4.3, 5.5)	4.9 (4.3, 5.5)
LDL-C (mmol/L)	3.2 (2.6, 3.8)	3.1 (2.6, 3.7)	3.2 (2.7, 3.7)	3.2 (2.7, 3.7)
HDL-C (mmol/L)	1.2 (1.0, 1.4)	1.2 (1.0, 1.4)	1.2 (1.0, 1.4)	1.2 (1.0, 1.4)
Triglyceride (mmol/L)	1.5 (1.1, 2.0)	1.5 (1.1, 2.0)	1.5 (1.1, 2.1)	1.5 (1.1, 2.1)
ALT (U/L)	22 (16, 31)	20 (15, 28)	21 (16, 30)	21 (16, 31)
AST (U/L)	22 (19, 26)	21 (17, 26)	22 (18, 27)	21 (17, 27)
Hemoglobin (g/L)	143 (134, 153)	141 (132, 151)	143 (134, 153)	141 (132, 153)
HOMA-IR	2.4 (1.6, 3.5)	2.4 (1.7, 3.6)	2.4 (1.6, 3.4)	2.5 (1.6, 3.5)
HOMA-*β*	77.3 (51.4, 109.6)	78.0 (51.5, 119.5)	78.1 (50.0, 115.8)	81.9 (52.8, 112.4)
Cytokines				
CRP (*μ*mol/L)	1.1 (0.6, 2.3)	1.1 (0.7, 2.4)	1.2 (0.7, 2.4)	1.3 (0.7, 2.4)
SOD	6.9 (2.0, 10.5)	6.8 (4.2, 10.0)	6.7 (4.0, 10.2)	7.0 (2.2, 11.1)
Amylin	7.6 (6.4, 9.5)	7.4 (6.4, 9.2)	7.7 (6.6, 9.5)	7.6 (6.4, 9.5)
IL-6	2.3 (1.5, 4.3)	2.3 (1.5, 4.0)	2.4 (1.5, 4.2)	2.3 (1.5, 3.6)
Urine albumin/Cr (mg/g)	6.9 (4.3, 14.5)	7.5 (4.5, 14.5)	7.9 (4.6, 15.3)	7.3 (4.4, 13.9)
Daily calorie intake (kcal/day)	1503 (1222, 1841)	1548 (1263, 1909)	1535 (1278, 1929)	1564 (1233, 1931)
Proportion of total calorie intake from carbohydrate (%)	61 (51, 69)	59 (49, 66)	60 (52, 68)	59 (50, 67)
Proportion of total calorie intake from protein (%)	14 (12, 17)	14 (12, 17)	14 (12, 16)	14 (12, 17)
Proportion of total calorie intake from fat (%)	23 (16, 30)	24 (19, 31)	24 (17, 31)	24 (17, 31)
Physical activity				
Low	109 (23)	116 (25)	123 (26)	108 (23)
Medium	244 (53)	255 (54)	239 (50)	246 (53)
High	111 (24)	99 (21)	112 (24)	114 (24)

**Table 2:** Proportions of individual developing diabetes and reverting back to normoglycemic state at 1, 2, and 3 years of follow-up, by treatment groups and the odds ratio (95% CI) of the likelihood of reverting to normoglycemic state and developing diabetes during 3 years of follow-up in the three treatment groups, compared to the conventional+placebo group.

	Conventional lifestyle+placebo	Intensive lifestyle+placebo	Conventional lifestyle+pioglitazone	Intensive lifestyle+pioglitazone
Year 1				
*N*	480	490	492	483
Lost to follow-up	88 (18.3)	92 (18.8)	95 (19.3)	91 (18.8)
DM	54 (11.3)	57 (11.6)	48 (9.8)	36 (7.5)
Normal	185 (38.5)	151 (30.8)	188 (38.2)	205 (42.4)
Year 2				
*N*	153	190	161	151
Lost to follow-up	51 (33.3)	70 (36.8)	68 (42.2)	43 (28.5)
DM	20 (13.1)	15 (7.9)	15 (9.3)	16 (10.6)
Normal	41 (26.8)	38 (20.0)	32 (19.9)	44 (29.1)
Year 3				
*N*	41	67	46	48
Lost to follow-up	18 (43.9)	31 (46.3)	18 (39.1)	25 (52.1)
DM	1 (2.4)	8 (11.9)	9 (19.6)	2 (4.2)
Normal	10 (24.4)	11 (16.4)	3 (6.5)	6 (12.5)
Overall				
Normal	236 (60.0)	200 (50.3)	224 (56.6)	255 (65.1)
SHR (95% CI)	Ref	0.61 (0.41, 0.93)	0.82 (0.55, 1.22)	1.15 (0.66, 1.57)
p		0.020	1.00	0.76
DM	76 (11.63)	80 (16.3)	72 (14.4)	54 (11.2)
SHR (95% CI)	Ref	1.00 (0.61, 1.57)	0.83 (0.47, 1.36)	0.55 (0.32, 0.90)
*p*		0.86	0.26	0.024

**Table 3:** Mean change (95% CI) or median change (95% CI of median) ^∗^ in the levels of secondary study parameters at 3 years of follow-up in the 4 treatment groups. The analysis is based on intention-to-treat analysis.

	Conventional lifestyle+placebo	Intensive lifestyle+placebo	Conventional lifestyle+pioglitazone	Intensive lifestyle+pioglitazone
*N* FPG change	23 -0.20 (-0.05, 0.10)	37 0.04 (-0.20, 0.28)	28 0.08 (-0.20, 0.35)	25 0.05 (-0.24, 0.34)
*N* PPG change	23 -1.05 (-2.0, -0.12)	37 -0.05 (-0.78, 0.69)	28 0.83 (-0.22, 1.67)	25 -0.92 (-0.81, -0.62)
*N* HbA1c change	27 0.30 (0.14, 0.46)	43 0.21 (0.09, 0.34)	32 0.20 (0.05, 0.34)	30 0.20 (0.05, 0.35)
*N* Weight change	24 -2.21 (-3.86, -0.56)	39 -1.06 (-2.40, 0.22)	28 -0.37 (-0.21, 0.94)	24 -1.35 (-3.00, 0.29)
*N* Waist change	24 -3.74 (-6.14, -1.33)	39 -0.95 (-2.83, 0.94)	27 -1.05 (-3.31, 1.21)	24 -2.45 (-4.56, -0.05)
*N* SBP change	24 -2 (-8, -3)	39 -3 (-7, 1)	28 0.3 (-5, 5)	24 -3 (-9, 2)
*N* LDL change	27 0.29 (-0.01, 0.60)	43 0.12 (-0.12, 0.36)	32 0.11 (-0.17, 0.38)	30 0.19 (-0.10, 0.47)
*N* HDL change	27 0.12 (0.04, 0.21)	43 0.17 (0.09, 0.24)	32 0.09 (-0.01, 0.17)	30 0.24 (0.16, 0.33)
*N* Triglyceride change^∗^	27 -0.12 (-0.41, 0.13)	43 -0.17 (-0.42, 0.20)	32 -0.03 (-0.28, 0.14)	30 -0.40 (-0.62, -0.13)
*N* hsCRP change^∗^	27 -0.10 (-0.34, 0.40)	43 -0.20 (-0.53, -0.02)	32 -0.08 (-0.40, 0.27)	30 -0.10 (-0.40, 0.19)
*N* C-peptide change^∗^	33 -0.50 (-0.67, -0.31)	53 -0.47 (-0.55, -0.37)	36 -0.31 (-0.72, -0.25)	39 -0.65 (-0.78, -0.31)
*N* Urine ACR change^∗^	27 -2.11 (-12.27, -0.99)	43 -1.74 (-3.12, 0.32)	31 -0.07 (-3.74, 4.70)	30 -4.00 (-7.56, -1.24)

## Data Availability

The data used to support the findings of this study may be accessed on request through the BPRP study group by contacting at the corresponding author of this manuscript.

## Abbreviations

ACR: Albumin/creatinine
ADD: Antidiabetes drug
BMI: Body mass index
BPRP: Beijing Prediabetes Reversion Program
CRP: C-reactive protein
CVD: Cardiovascular disease
DPP: Diabetes Prevention Program
DPS: Finnish Diabetes Prevention Study
HbA1c: Hemoglobin A1c
HDL-C: HDL-cholesterol
HOMA-beta: Homeostatic model assessment for beta cell function
HOMA-IR: Homeostatic model assessment for insulin resistance
IFG: Impaired fasting glucose
IGT: Impaired glucose tolerance
IL-6: Interleukin-6
LDL-C: LDL-cholesterol
OGTT: Oral glucose tolerance test
PG: Plasma glucose
SOD: Superoxide dismutase
TC: Total cholesterol
TG: Triglyceride
TZD: Thiazolidinedione.

## Additional Points

*Study Sites and Principle Investigator of Each Site*. Beijing Tongren Hospital: Jingkui Yang. Beijing Hospital: Lixin Guo. The Military General Hospital of Beijing PLA: Xiaofeng Lv. Peking University First Hospital: Xiaohui Guo. Peking Union Medical Colledge Hospital: Hongding Xiang. Fuxing Hospital: Xiaoming Zhuang. Beijing No.6 Hospital: Shangnong Wang. The Second Artillery General Hospital of Chinese People's Liberation Army: Quanmin Li. Beijing Zhanlanlu Hospital: Shuling Chang. Peking University Third Hospital: Tianpei Hong. Chinese PLA General Hospital: Juming Lu. The 304th Hospital: Shinan Yin. The 309th Hospital of Chinese People's Liberation Army: Yan Zhang. Navy General Hospital: Qiyu Guo. Air Force General Hospital: Xiaohong Guan. Beijing Haidian Hospital: Wei Huang. China-Japan Friendship Hospital: Guangwei Li. The 306th Hospital of PLA: Zhangrong Xu. Beijing Chaoyang Hospital: Yuan Xu. Beijing Chuiyangliu Hospital: Cuiping Liu. China Meitan General Hospital: Hongmei Li. Civil Aviation General Hospital: Dingqiong Peng. Xuanwu Hospital: Li Wang. Beijing Tiantan Hospital: Lirong Zhong. Aerospace 731 Hospital: Yuming Liu. Beijing Electric Power Hospital: Dongmei Ni. The Luhe Teaching Hospital of the Capital Medical University: Dong Zhao. People's Hospital of Beijing Daxing District: Changchun Xue. Beijing Pinggu Hospital: Yufeng Li. Daxing Hospital of Tranditional Chinese Medicine: Li Ma. Beijing Chaoyang Hospital (Jingxi Campus): Shan Gao. Beijing Hepingli Hospital: Yaping Liu. Peking University Shougang Hospital: Xiaoping Lu. Nanyuan Hospital: Kaijie Yang. Beijiao Hospital: Z. Jing. Nanfaxin Hospital: Rongmin Zhang.

## Supplementary Materials

Supplementary 1 Supplement Figure 1: compliance of the lifestyle intervention in intensive lifestyle intervention groups.

Supplementary 2 Supplement Table 1: adverse events which were considered maybe relevant to treatment by investigators among four groups.

## References

[1] Wang L., Gao P., Zhang M. (2017). Prevalence and ethnic pattern of diabetes and prediabetes in China in 2013. *JAMA*.

[2] Grundy S. M. (2012). Pre-diabetes, metabolic syndrome, and cardiovascular risk. *Journal of the American College of Cardiology*.

[3] Harris M. I. (1996). Impaired glucose tolerance--prevalence and conversion to NIDDM. *Diabetic Medicine*.

[4] Pan X. R., Li G. W., Hu Y. H. (1997). Effects of diet and exercise in preventing NIDDM in people with impaired glucose tolerance. The Da Qing IGT and Diabetes Study. *Diabetes Care*.

[5] Gong Q., Zhang P., Wang J. (2016). Changes in mortality in people with IGT before and after the onset of diabetes during the 23-year follow-up of the Da Qing Diabetes Prevention Study. *Diabetes Care*.

[6] Ibrahim M., Tuomilehto J., Aschner P. (2018). Global status of diabetes prevention and prospects for action: a consensus statement. *Diabetes/Metabolism Research and Reviews*.

[7] Knowler W. C., Fowler S. E., Hamman R. F. (2009). 10-year follow-up of diabetes incidence and weight loss in the Diabetes Prevention Program Outcomes Study. *Lancet*.

[8] Lindstrom J., Louheranta A., Mannelin M. (2003). The Finnish Diabetes Prevention Study (DPS): lifestyle intervention and 3-year results on diet and physical activity. *Diabetes Care*.

[9] Lindstrom J., Ilanne-Parikka P., Peltonen M. (2006). Sustained reduction in the incidence of type 2 diabetes by lifestyle intervention: follow-up of the Finnish Diabetes Prevention Study. *Lancet*.

[10] Chiasson J. L., Josse R. G., Gomis R., Hanefeld M., Karasik A., Laakso M. (2002). Acarbose for prevention of type 2 diabetes mellitus: the STOP-NIDDM randomised trial. *Lancet*.

[11] Gerstein H. C., Yusuf S., Bosch J. (2006). Effect of rosiglitazone on the frequency of diabetes in patients with impaired glucose tolerance or impaired fasting glucose: a randomised controlled trial. *Lancet*.

[12] DeFronzo R. A., Tripathy D., Schwenke D. C. (2011). Pioglitazone for diabetes prevention in impaired glucose tolerance. *The New England Journal of Medicine*.

[13] Ramachandran A., Snehalatha C., Mary S., Mukesh B., Bhaskar A. D., Vijay V. (2006). The Indian Diabetes Prevention Programme shows that lifestyle modification and metformin prevent type 2 diabetes in Asian Indian subjects with impaired glucose tolerance (IDPP-1). *Diabetologia*.

[14] Knowler W. C., Barrett-Connor E., Fowler S. E. (2002). reduction in the incidence of type 2 diabetes with lifestyle intervention or metformin. *The New England Journal of Medicine*.

[15] Holman R. R., Coleman R. L., Chan J. C. N. (2017). Effects of acarbose on cardiovascular and diabetes outcomes in patients with coronary heart disease and impaired glucose tolerance (ACE): a randomised, double-blind, placebo-controlled trial. *The Lancet Diabetes and Endocrinology*.

[16] Knowler W. C., Hamman R. F., Edelstein S. L. (2005). prevention of type 2 diabetes with troglitazone in the Diabetes Prevention Program. *Diabetes*.

[17] Haw J. S., Galaviz K. I., Straus A. N. (2017). Long-term sustainability of diabetes prevention approaches: a systematic review and meta-analysis of randomized clinical trials. *JAMA Internal Medicine*.

[18] Luo Y., Paul S. K., Zhou X. (2017). Rationale, design, and baseline characteristics of Beijing Prediabetes Reversion Program: a randomized controlled clinical trial to evaluate the efficacy of lifestyle intervention and/or pioglitazone in reversion to normal glucose tolerance in prediabetes. *Journal Diabetes Research*.

[19] Ji L., Hu D., Pan C. (2013). Primacy of the 3B approach to control risk factors for cardiovascular disease in type 2 diabetes patients. *The American Journal of Medicine*.

[20] Tuomilehto J., Lindstrom J., Eriksson J. G. (2001). prevention of type 2 diabetes mellitus by changes in lifestyle among subjects with impaired glucose tolerance. *The New England Journal of Medicine*.

[21] Piller C. (2019). A war on "prediabetes" has created millions of new patients and a tempting opportunity for pharma. But how real is the condition?. *Science*.

