# Supplementary figures and images for: A Randomized Controlled Clinical Trial of Lifestyle Intervention and Pioglitazone for Normalization of Glucose Status in Chinese with Prediabetes
Source: J Diabetes Res. 2022 Jan 6;2022:2971382. doi: 10.1155/2022/2971382 (PMC8759441; doi:10.1155/2022/2971382)

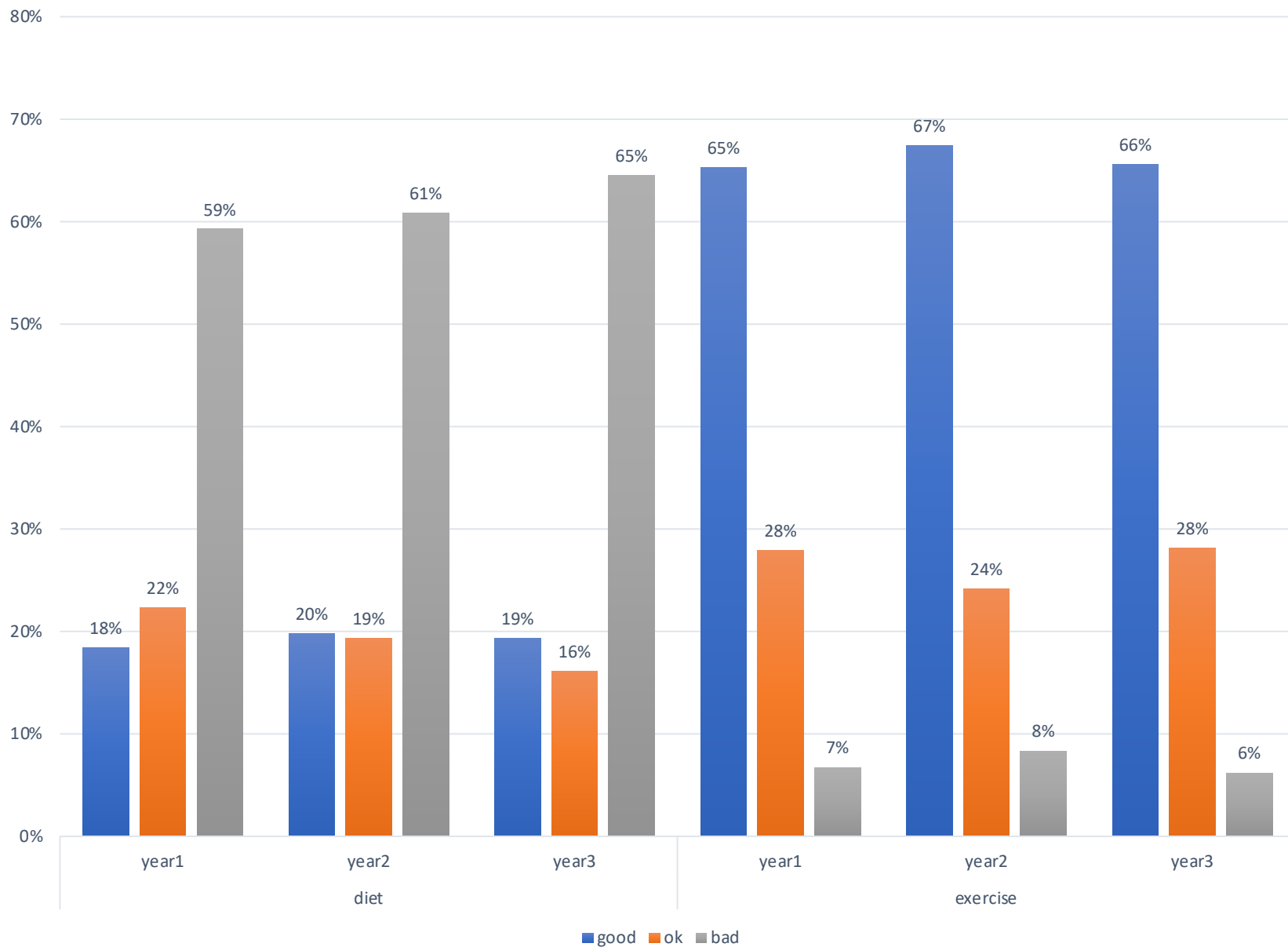

Supplement: Supplementary 1 — Supplement Figure 1: compliance of the lifestyle intervention in intensive lifestyle intervention groups. [file 2971382.f1.pdf]
