# Supplementary material for: A Randomized Controlled Clinical Trial of Lifestyle Intervention and Pioglitazone for Normalization of Glucose Status in Chinese with Prediabetes
Source: J Diabetes Res. 2022 Jan 6;2022:2971382. doi: 10.1155/2022/2971382 (PMC8759441; doi:10.1155/2022/2971382)
Supplement: Supplementary 2 — Supplement Table 1: adverse events which were considered maybe relevant to treatment by investigators among four groups. [file 2971382.f2.docx]

Supplement table 1. Adverse events which were considered maybe relevant to treatment by investigators among four groups

|  | **Total** | **Conventional**  **and placebo** | **Intensive**  **and placebo** | **Conventional**  **and pioglitazone** | **Intensive**  **and pioglitazone** | **P^*^** |
| --- | --- | --- | --- | --- | --- | --- |
| Edema | 34 (1.7%) | 13 (2.7%) | 5 (1.0%) | 8 (1.6%) | 8 (1.7%) | 0.268 |
| Palpitation | 17 (0.9%) | 3 (0.6%) | 4 (0.8%) | 7 (1.4%) | 3 (0.6%) | 0.572 |
| Dizzy | 15 (0.8%) | 5 (1.0%) | 3 (0.6%) | 5 (1.0%) | 2 (0.4%) | 0.631 |
| Abnormal liver function | 15 (0.8%) | 3 (0.6%) | 4 (0.8%) | 3 (0.6%) | 5 (1.0%) | 0.857 |
| Osteoarthrosis | 13 (0.7%) | 5 (1.0%) | 2 (0.4%) | 3 (0.6%) | 3 (0.6%) | 0.657 |
| Hypoglycemia symptom | 11 (0.6%) | 2 (0.4%) | 4 (0.8%) | 3 (0.6%) | 2 (0.4%) | 0.894 |
| Leukopenia | 10 (0.5%) | 2 (0.4%) | 1 (0.2%) | 6 (1.2%) | 1 (0.2%) | 0.143 |
| Headache | 10 (0.5%) | 0 | 3 (0.6%) | 5 (1.0%) | 2 (0.4%) | 0.165 |
| Rash | 9 (0.5%) | 3 (0.6%) | 1 (0.2%) | 3 (0.6%) | 2 (0.4%) | 0.747 |
| Hypertension | 8 (0.4%) | 3 (0.6%) | 1 (0.2%) | 1 (0.2%) | 3 (0.6%) | 0.566 |
| Cold symptoms | 5 (0.3%) | 1 (0.2%) | 2 (0.4%) | 2 (0.4%) | 0 | 0.735 |
| Shortness of breath | 3 (0.2%) | 0 | 0 | 3 (0.6%) | 0 | 0.062 |
| Constipation | 3 (0.2%) | 0 | 0 | 2 (0.4%) | 1 (0.2%) | 0.528 |
| Fatigue | 3 (0.2%) | 1 (0.2%) | 0 | 2 (0.4%) | 0 | 0.480 |
| Backache | 3 (0.2%) | 0 | 3 (0.6%) | 0 | 0 | 0.046 |
| Gastritis | 3 (0.2%) | 1 (0.2%) | 0 | 1 (0.2%) | 1 (0.2%) | 0.810 |
| Abdominal pain | 2 (0.1%) | 0 | 1 (0.2%) | 1 (0.2%) | 0 | 1.000 |
| Numbness | 2 (0.1%) | 2 (0.4%) | 0 | 0 | 0 | 0.061 |
| Tinnitus | 2 (0.1%) | 1 (0.2%) | 0 | 1 (0.2%) | 0 | 0.622 |
| Diarrhea | 2 (0.1%) | 1 (0.2%) | 1 (0.2%) | 0 | 0 | 0.497 |
| Urinary infection | 2 (0.1%) | 0 | 0 | 2 (0.4%) | 0 | 0.250 |
| Anemia | 2 (0.1%) | 1 (0.2%) | 0 | 1 (0.2%) | 0 | 0.622 |
| Premature beat | 2 (0.1%) | 1 (0.2%) | 1 (0.2%) | 0 | 0 | 0.497 |
| Clporrhagia | 1 (0.1%) | 1 (0.2%) | 0 | 0 | 0 | 0.247 |
| Gynecological inflammation | 1 (0.1%) | 1 (0.2%) | 0 | 0 | 0 | 0.247 |
| Abnormal ECG | 1 (0.1%) | 0 | 0 | 1 (0.2%) | 0 | 1.000 |
| Elevated BUN | 1 (0.1%) | 0 | 1 (0.2%) | 0 | 0 | 0.747 |
| Abdominal distention | 1 (0.1%) | 0 | 1 (0.2%) | 0 | 0 | 0.747 |
| Cough | 1 (0.1%) | 1 (0.2%) | 0 |  | 0 | 0.247 |
| Macular degeneration | 1 (0.1%) | 0 | 0 | 0 | 1 (0.2%) | 0.495 |
| Muscle soft tissue injury | 1 (0.1%) | 0 | 0 | 0 | 1 (0.2%) | 0.495 |
| Anxiety and depression | 1 (0.1%) | 0 | 0 | 1 (0.2%) | 0 | 1.000 |
| Conjunctivitis | 1 (0.1%) | 1 (0.2%) | 0 | 0 | 0 | 0.247 |
| Granulocytopenia | 1 (0.1%) | 0 | 0 | 0 | 1 (0.2%) | 0.495 |
| Cerebral circulation insufficiency | 1 (0.1%) | 0 | 1 (0.2%) | 0 | 0 | 0.747 |
| Cerebral vascular disease | 1 (0.1%) | 0 | 0 | 0 | 1 (0.2%) | 0.495 |
| Skin itch | 1 (0.1%) | 0 | 1 (0.2%) | 0 | 0 | 0.747 |
| Gout | 1 (0.1%) | 0 | 0 | 0 | 1 (0.2%) | 0.495 |
| Hair loss | 1 (0.1%) | 1 (0.2%) | 0 | 0 | 0 | 0.247 |
| Injury | 1 (0.1%) | 0 | 0 | 1 (0.2%) | 0 | 1.000 |
| Peptic ulcer | 1 (0.1%) | 0 | 0 | 1 (0.2%) | 0 | 1.000 |
| Sexual dysfunction | 1 (0.1%) | 1 (0.2%) | 0 | 0 | 0 | 0.247 |
| Thrombocytopenia | 1 (0.1%) | 0 | 0 | 1 (0.2%) | 0 | 1.000 |
| Toothache | 1 (0.1%) | 0 | 0 | 1 (0.2%) | 0 | 1.000 |
| Periodontitis | 1 (0.1%) | 0 | 0 | 1 (0.2%) | 0 | 1.000 |
| Eye disease | 1 (0.1%) | 0 | 1 (0.2%) | 0 | 0 | 0.747 |
| Acne | 1 (0.1%) | 0 | 0 | 1 (0.2%) | 0 | 1.000 |

***Fisher exact test was used to compare the difference among groups**
